# Improving membrane protein expression and function using genomic edits

**DOI:** 10.1038/s41598-017-12901-7

**Published:** 2017-10-12

**Authors:** Heather M. Jensen, Thomas Eng, Victor Chubukov, Robin A. Herbert, Aindrila Mukhopadhyay

**Affiliations:** 1 Joint BioEnergy Institute, Emeryville, CA 94608 USA; 2 Biological Systems and Engineering Division, Lawrence Berkeley National Laboratory, Berkeley, CA 94720 USA

## Abstract

Expression of membrane proteins often leads to growth inhibition and perturbs central metabolism and this burden varies with the protein being overexpressed. There are also known strain backgrounds that allow greater expression of membrane proteins but that differ in efficacy across proteins. We hypothesized that for any membrane protein, it may be possible to identify a modified strain background where its expression can be accommodated with less burden. To directly test this hypothesis, we used a bar-coded transposon insertion library in tandem with cell sorting to assess genome-wide impact of gene deletions on membrane protein expression. The expression of five membrane proteins (CyoB, CydB, MdlB, YidC, and LepI) and one soluble protein (GST), each fused to GFP, was examined. We identified *Escherichia coli* mutants that demonstrated increased membrane protein expression relative to that in wild type. For two of the proteins (CyoB and CydB), we conducted functional assays to confirm that the increase in protein expression also led to phenotypic improvement in function. This study represents a systematic approach to broadly identify genetic loci that can be used to improve membrane protein expression, and our method can be used to improve expression of any protein that poses a cellular burden.

## Introduction

Membrane proteins are critical to cellular functions such as response to environmental changes, membrane stability, nutrient transport, redox balance, energy generation, and cellular defense. However, cells must carefully balance the necessary functions of many membrane proteins against the capacity to produce and translocate membrane proteins maintaining membrane physiology. This is evidenced by many bacterial studies^1–3^ showing that overexpression of membrane proteins tends to have far stronger deleterious effects on cell health than similar overexpression of cytosolic proteins. Hypotheses that have been proposed to explain this phenomenon include the overutilization of membrane translocation systems or chaperones, and alterations in central metabolism revolving around respiration^1,4,5^, but no unifying theory has emerged.

The deleterious effect of membrane protein overexpression and the relatively poor understanding of the underlying cell biology translates into significant challenges in biological engineering. Membrane proteins (and complexes) are frequent targets for cellular engineering applications, such as membrane-spanning electron conduits^6,7^, responses to environmental cues^8,9^, and transport of nutrients or products during bioproduction^10–14^. Additionally, biochemical and structural studies of membrane proteins require expression of large amounts of the protein in bacterial cells, and these studies are hampered by the deleterious effects of expression on cell growth. Current approaches to improving membrane protein expression largely use tighter transcriptional control to minimize the effect on cell viability^15–18^. These approaches require significant optimization of growth conditions, inducer concentration, and precise control of plasmid copy number^5,18^. Some efforts have been made to modify the host cell to better accommodate membrane protein expression, for instance, through chaperone coexpression^19–21^, maintenance of respiratory metabolism^22^, or genetic selection^15,23,24^. Most recently the BL21(DE3) strain commonly used for greater membrane protein expression^15,25^ was improved via an additional mutation in the T7 RNAP^26^.

In this study, we developed an unbiased high-throughput sequencing-based method that permits concurrent analysis of the effect of every non-essential *Escherichia coli* gene on membrane protein expression. We hypothesized that under routine laboratory cultivation conditions, many non-essential proteins are potentially antagonistic to the expression of a desired membrane protein. Such an impact, either direct or indirect, would only occur during expression of the targeted protein. We anticipated that a genome-wide search for gene disruptions that allow increased membrane protein expression would lead to the identification of superior microbial host chasses for the expression of a desired membrane protein.

To identify such microbial host chasses, we used a pooled, bar-coded transposon library (TnLib)^27,28^ in *E*. *coli* BW25113 consisting of ~150,000 unique transposon insertion strains to screen for genetic alterations that lessen the burden of plasmid-borne membrane protein expression. We used this transposon library to interrogate five inner membrane protein (IMP) complexes (CyoB, CydB, MdlB, YidC, and LepI) which had distinct protein functions (Table 1) with GFP intensity as a proxy for increased protein expression. CyoABCD is a cytochrome *bo* terminal oxidase expressed under high oxygen conditions; CydAB is a cytochrome *bd*-I terminal oxidase expressed under oxygen-limited conditions^29,30^. MdlB is an ABC efflux pump that confers microbial tolerance to the five carbon alcohol, isopentenol (3-methyl-3-buten-1-ol)^31^. YidC is a chaperone that mediates the insertion and assembly of inner membrane proteins in association with the Sec translocon^32,33^. LepI is a modified version of leader peptidase (LepB) such that it inserts in the inner membrane with inverted topology and is thus nonfunctional^34,35^. We also selected one cytoplasmic protein (GST, glutathione *S*-transferase) for analysis of soluble recombinant protein expression.

**Table 1:** Transposon libraries and membrane proteins used in this study.

TnLib	IMP-GFP	Gene(s) expressed	Function	Plasmid Alias	Plasmid Name	TnLib diversity^1^
TnLib/*cyoB-GFP*	CyoB-GFP	*cyoAB*_*GFP*_ *CD*	Aerobic cytochrome *bo* terminal oxidase	*/cyoB-GFP*	pHJ-050	98.9%
TnLib/*cydB-GFP*	CydB-GFP	*cydAB*_*GFP*_	Micro-aerobic cytochrome *bd* terminal oxidase	*/cydB-GFP*	pHJ-051	96.9%
TnLib/*mdlB-GFP*	MdlB-GFP	*mdlB*_*GFP*_	ABC transporter	*/mdlB-GFP*	pHJ-055	97.6%
TnLib/*yidC-GFP*	YidC-GFP	*yidC*_*GFP*_	Inner membrane protein insertion factor	*/yidC-GFP*	pHJ-066	97.1%
TnLib/*lepI-GFP*	LepI-GFP	*lepI*_*GFP*_	Non-functional IMP control: Inverted leader peptidase	*/lepI-GFP*	pHJ-052	98.2%
TnLib/*gstA-GFP*	GST-GFP	*gstA*_*GFP*_	Soluble protein control	*/gstA-GFP*	pHJ-053	98.8%

^1^Diversity is reported as the number of bar codes that are observed at least twice after transformation with the plasmid DNA with respect to the non-transformed TnLib.

In this study, we identified gene disruptions that increase membrane protein expression in *E*. *coli* BW25113. We explored trends in protein functional groups and the distribution of genome-wide gene deletions expressing different IMP complexes. Overexpression in candidate transposon mutants was verified for CyoABCD, CydAB, and MdlB by evaluating expression of the corresponding IMP in single-gene deletion knockout mutants. Lastly, we demonstrated improved functional expression of Cyo and Cyd complexes when expressed in our candidate deletion backgrounds. Our study provides a path for identifying the microbial-host chasses that are applicable for improved membrane protein expression.

## Results

### Experimental approach for the Identification of Genes Detrimental to IMP Expression

**Figure 1:**
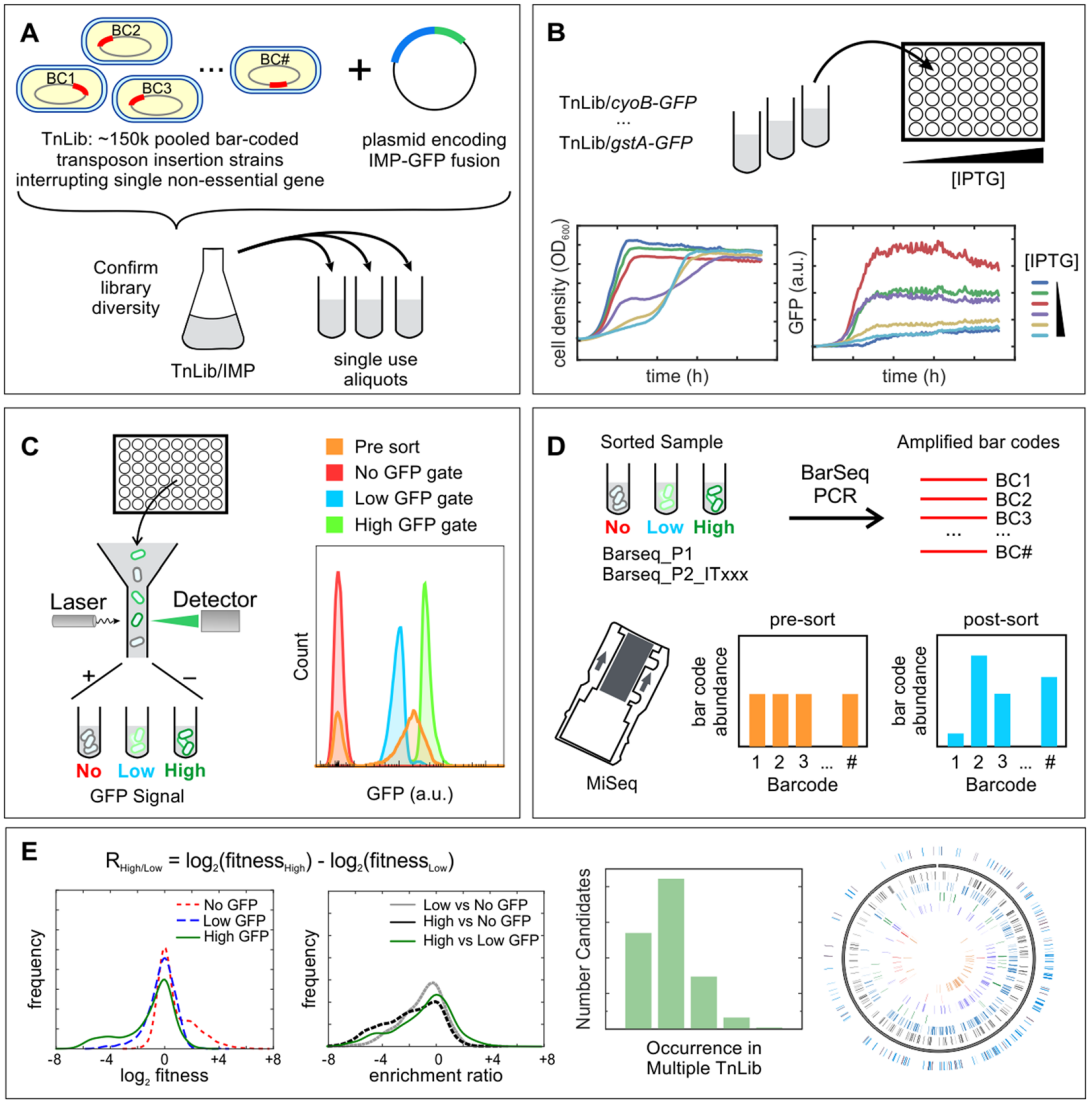
Workflow to determine genetic mutants detrimental to membrane protein expression. (**A**) The pooled, bar-coded *E*. *coli* transposon insertion library (TnLib) containing ~150k strains with single gene disruptions was transformed with a plasmid encoding an inner membrane protein GFP fusion (IMP-GFP), resulting in the transposon library TnLib/IMP. (BC = bar code) (**B**) Growth and expression of five IMP-GFP fusions were tested under a range of inducer concentration to find toxic growth conditions. (**C**) Fluorescence-activated cell sorting (FACS) isolated the unique bar-coded mutants grown in toxic membrane protein expression conditions into gates of no, low, or high GFP signal. (**D**) Bar codes of mutants enriched in sorted populations were amplified using BarSeq, and the abundance of each bar code was determined using next generation sequencing. (**E**) Enrichment ratios were used to determine preferential IMP-GFP expression associated with each gene disruption.

We screened all non-essential *E*. *coli* genes for their impact on protein expression in a high throughput manner. This systems-wide approach combined high-throughput, genome-wide evaluation of strain fitness with fluorescence-activated cell sorting (FACS) to identify genetic determinants detrimental to toxic protein expression (Fig. 1). First, we transformed a previously characterized *E*. *coli* transposon library^27^ with a plasmid encoding a candidate IMP with an in-frame superfolder green fluorescent protein (GFP) fusion joined with a linker (IMP-GFP, Table 1). Next, the libraries harboring IMP-GFP expression plasmids (TnLib/IMP) were grown under toxic IMP expression conditions and sorted based on IMP-GFP signal using FACS. The relative fitness of each bar-coded gene disruption in the sorted populations was determined using BarSeq^27^, and the fitness across different sorting gates was compared to determine the preferential IMP-GFP expression level of any gene disruption. We examined five selected IMP candidates and one cytoplasmic candidate (Table 1). A nonfunctional membrane protein, LepI, accounts for transposon insertions that generally impact IMP expression without downstream functional effects. Similarly, the cytoplasmic soluble protein, GST, distinguishes transposon insertions responsible for improving soluble protein expression.

To ensure the diversity of the TnLib/IMP is maintained after transformation with the IMP plasmid, we evaluated bar code occurrence of the transformed TnLib/IMP with BarSeq. 91.9% of known barcodes were observed at least twice in electrocompetent TnLib. Of those, greater than 97% are observed at least twice after transformation in all TnLib/IMP (Table 1), thus we conclude that library diversity is maintained in all TnLib/IMP. Growth rate and GFP intensity in whole culture were determined with respect to inducer (isopropyl β-D-1-thiogalactopyranoside, IPTG) concentration to find a condition that induces mild IMP expression stress without completely impeding growth. All TnLib/IMP growth rates decreased beyond a critical concentration of inducer, but TnLib and TnLib/*gstA-GFP* displayed constant growth rate with all IPTG concentrations tested (Fig. S1). Maximum GFP intensity in whole culture also decreased as induction of IMP expression reached toxic levels while TnLib/*gstA-GFP* showed a typical increase in GFP signal with increasing inducer concentration (Fig. S1). Flow cytometry was used to determine the GFP signal per cell in all TnLib/IMP (Fig. S2). The TnLib/ IMP GFP signal is unimodal with low inducer concentrations, but we observed a bimodal distribution of cells with and without GFP signal at high inducer concentrations (Fig. S2). Additionally, the population of the culture with a GFP signal decreases as inducer concentration increases (Fig. S2). In all subsequent experiments, we used the empirically determined inducer concentrations that cause a mild membrane protein expression stress response.

**Figure 2:**
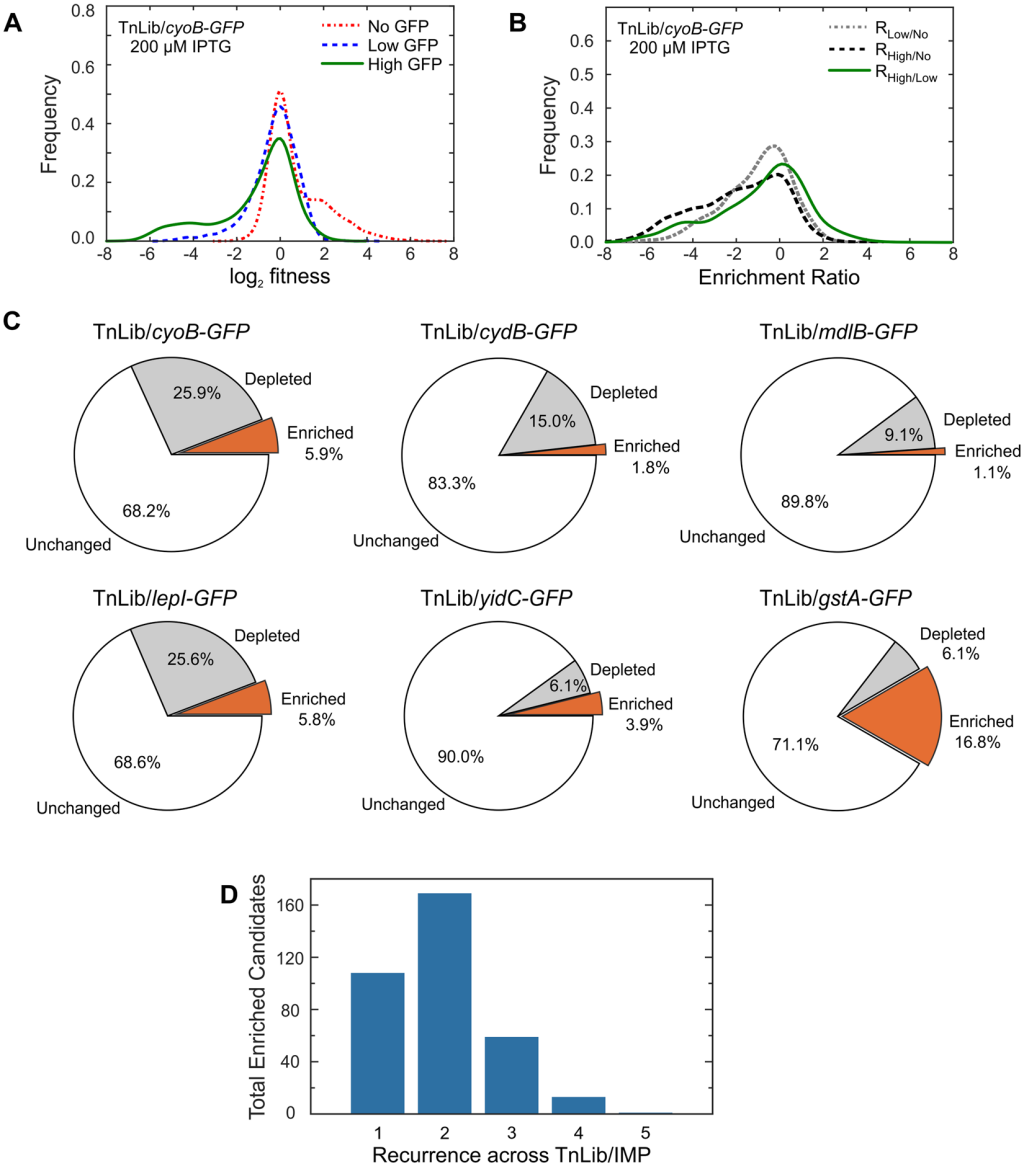
Overview of Method Workflow. (**A**) Representative distribution (TnLib/*cyoB-GFP*) of the relative fitness (log_2_ fitness) of gene disruption strains as determined from the relative abundance of transposon bar codes in sorted samples. (**B**) Representative distribution (TnLib/*cyoB-GFP*) of enrichment ratios, a comparison of the relative fitness of gene disruptions between different sorted gates. Gene disruptions with enrichment ratios above +2.0 or below −2.0 are considered enriched or depleted abundance, respectively. (**C**) Across all TnLib/IMP tested, 1–6% of bar-coded strains are enriched when Low GFP to High GFP sorted samples (R_High/Low_, orange wedge). (**D**) Among candidates that were found enriched in at least two biological replicates, 108 candidates are unique to a single TnLib/IMP and only 14 candidates were observed in at least four of the five TnLib/IMP tested. See also Fig. S3.

TnLib/IMP grown under membrane protein expression stress conditions were isolated using FACS based on IMP-GFP signal, a proxy for increased IMP expression per cell. Cells were sorted into one of three populations: No GFP, Low GFP, or High GFP. Genomic DNA was extracted from one million cells of each sorted population, and the bar codes were amplified by BarSeq PCR^27^. We determined the relative abundance bar codes in each sorted population using a previously described analysis method, FEBA^27^. Approximately ten unique transposon insertions represent each gene locus, and the total abundance of the corresponding ten bar codes were combined to create a metric of fitness (log_2_ fitness) for the gene. This metric represents the relative abundance of the gene disruption observed in the No GFP, Low GFP, and High GFP sorted populations (Figs 2A and S3, Table S1). A gene disruption that benefits IMP expression is represented by a positive log_2_ fitness value due to an increase in corresponding bar-coded strain abundance. Likewise, a gene disruption that is detrimental to IMP expression is represented by a negative log_2_ fitness value due to a decrease in corresponding bar-coded strain abundance.

### Comparative Analysis to Identify Transposon Insertion Mutants with High IMP-GFP Expression

We compared log_2_ fitness values of the sorted populations to identify strains able to express more of a desired IMP under expression stress conditions. These enrichment ratios (R_Low/No_, R_High/No_, and R_High/Low_) are the log_2_ ratios of the fitness values calculated from bar code abundances as described above (Fig. 2B, Fig. S3, Table S1). Enrichment ratios with values greater than +2.0 were considered enriched, and we infer that the corresponding gene disruption benefits IMP expression (for example, the R_High/Low_ for *oppF*, b1247 for CyoB-GFP at 200 μM IPTG is 5.615, Table S1). The relatively small number of cells sequenced in each experiment meant that a large number of genes had very few reads, thus creating a large number of potential false depleted hits. We thus only analyzed mutants with enriched abundance, which we interpreted as gene disruptions beneficial to the overproduction of the desired IMP complex. For each library, 1–6% of bar-coded strains were enriched in R_High/Low_ (Fig. 2C). Fewer enriched candidates were observed in R_High/No_ and R_Low/No_ (0.4–1.0% and 0.3–0.7%, Fig. S3 and Table S1).

**Figure 3:**
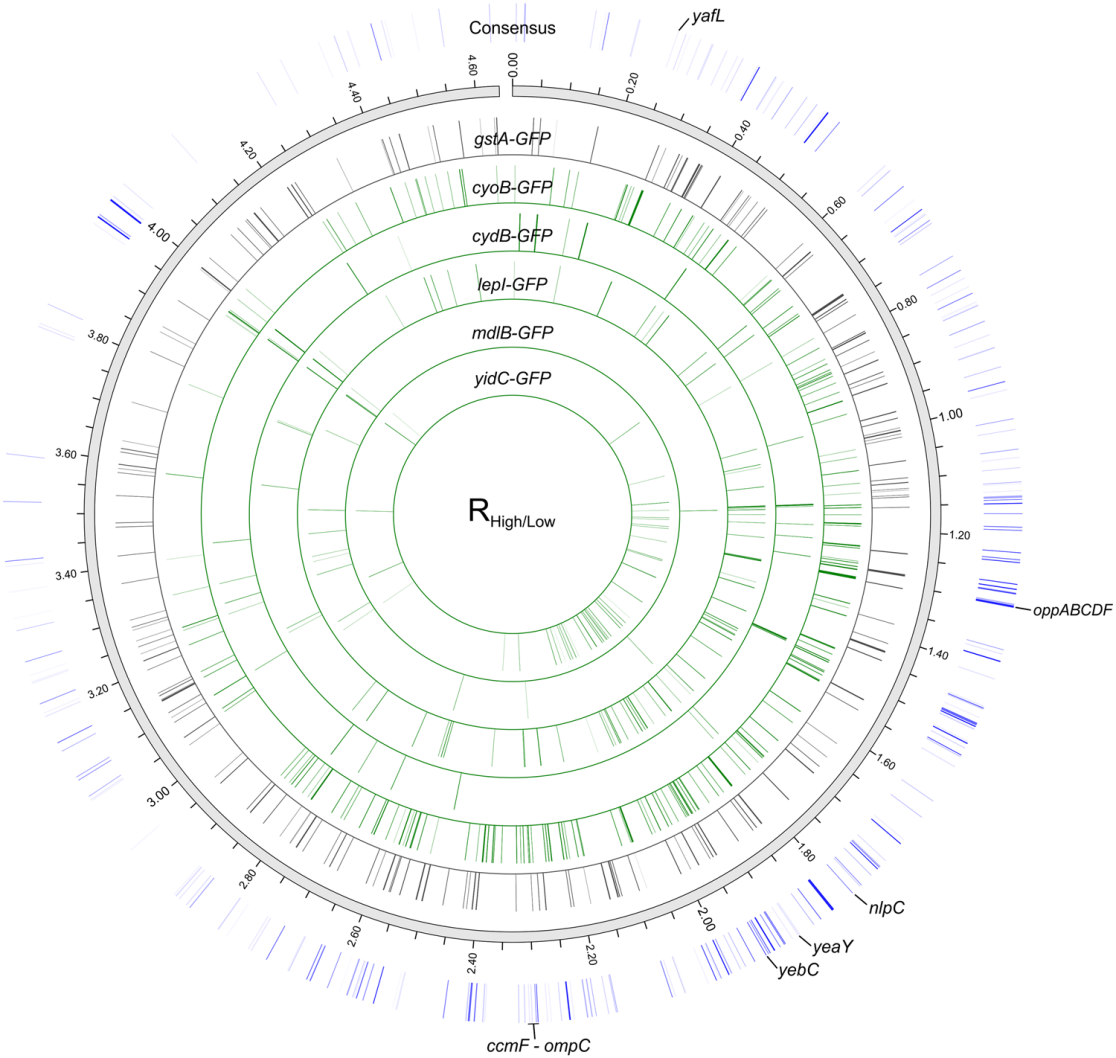
Genome-wide view of single gene disruptions enriched in high GFP populations. Gene disruptions enriched in at least two biological replicates (R_High/Low_, n ≥ 2) are shown for TnLib/*gstA-GFP* (grey inner band) and all TnLib/IMP tested (green inner bands). The consensus band (blue outer band) represents gene disruptions enriched in at least three replicates across all TnLib/IMP, for which color density increases with instances of enrichment.

Biological replicates of uninduced sorted samples were compared to determine the percentage of false positives (see Methods) and indicated that we can expect 20–30% of enriched gene disruptions to be false hits (Fig. S4). This variation may be attributed to low genomic template concentration in BarSeq but can be overcome using biological replicates for each TnLib/IMP to find bar-coded strains that are consistently enriched in the High GFP sorted population. The top 100 transposon mutant candidates with enrichment ratios greater than + 2.0 that were observed in at least 2 replicates for a given TnLib/IMP are listed in Table 2, and the data for all enriched candidates are listed in Table S2. These candidates represent genes potentially detrimental to recombinant IMP expression. To discern if the enrichment was similar or distinct between the libraries expressing different IMPs, we determined the frequency of the enriched candidates (R_High/Low_ ≥ 2.0, n ≥ 2) across the different libraries tested (Fig. 2D and Table S2). About 30% of enriched candidates are unique to a single TnLib/IMP, and about half were similar between at least two libraries. Only 14 candidates were enriched in at least four of the five libraries expressing a membrane protein (Fig. 2D and Table 2). For a genome-wide view of the gene disruptions impacting IMP expression, enriched candidates were mapped to their genomic loci (Fig. 3). Each bar represents a gene disruption enriched in at least two replicates of a given TnLib/IMP (Fig. 3, inner bands). The bars in the consensus band represent interrupted genes enriched in at least 3 replicates across all TnLib/IMP, with the color density representing increasing number of enriched replicates (Fig. 3, blue outer band). The region with the highest density of enriched genes for all TnLib/IMP was 1.0–2.1 Mb (Fig. 3, blue outer band).

**Table 2:** Top 100 enriched gene disruptions beneficial to IMP expression identified.

Rank	Gene	Number of enriched replicates in each libarary (TnLib/…)						Overall Number of Replicates		Observed in Number of Libraries	
		*cyoB-GFP*	*cydB-GFP*	*lepI-GFP*	*mdlB-GFP*	*yidC-GFP*	*gstA*-GFP	Total replicates	IMP-only replicates	Total frequency	IMP frequency
1	*hflD*	5	2	2	0	2	0	11	11	4	4
2	*dadX*	5	1	1	2	1	1	11	10	6	5
3	*rep*	3	3	2	0	2	0	10	10	4	4
4	*ybeZ*	5	2	2	1	0	0	10	10	4	4
5	*yceD*	5	1	2	0	2	0	10	10	4	4
6	*astD*	5	0	2	0	2	1	10	9	4	3
7	*proQ*	5	0	2	0	2	0	9	9	3	3
8	*ydhZ*	5	1	2	0	1	0	9	9	4	4
9	*yeeW*	5	0	2	0	2	2	11	9	4	3
10	*aldA*	4	2	1	1	0	0	8	8	4	4
11	*tatA*	2	0	2	2	2	1	9	8	5	4
12	*cfa*	5	0	2	0	1	1	9	8	4	3
13	*edd*	5	0	2	0	1	2	10	8	4	3
14	*fabF*	5	0	2	0	1	0	8	8	3	3
15	*fliN*	5	0	2	0	1	0	8	8	3	3
16	*hspQ*	5	0	2	0	1	1	9	8	4	3
17	*nlpC*	3	2	2	1	0	0	8	8	4	4
18	*oppC*	5	0	2	0	1	0	8	8	3	3
19	*oppD*	5	0	2	0	1	0	8	8	3	3
20	*pspE*	4	1	2	0	1	0	8	8	4	4
21	*ybgE*	5	1	2	0	0	0	8	8	3	3
22	*yedE*	5	0	2	0	1	2	10	8	4	3
23	*yedI*	5	1	2	0	0	0	8	8	3	3
24	*astA*	5	0	2	0	0	2	9	7	3	2
25	*ccmA*	5	0	2	0	0	1	8	7	3	2
26	*cheZ*	5	1	1	0	0	0	7	7	3	3
27	*cysA*	5	0	2	0	0	0	7	7	2	2
28	*dinI*	5	0	1	1	0	2	9	7	4	3
29	*hpt*	2	3	2	0	0	0	7	7	3	3
30	*htpG*	5	0	2	0	0	1	8	7	3	2
31	*mhpT*	5	1	1	0	0	0	7	7	3	3
32	*minD*	0	3	1	1	2	1	8	7	5	4
33	*narJ*	5	1	1	0	0	1	8	7	4	3
34	*nfo*	5	0	2	0	0	1	8	7	3	2
35	*oppF*	5	0	2	0	0	1	8	7	3	2
36	*tyrR*	3	1	2	0	1	0	7	7	4	4
37	*uidC*	4	1	2	0	0	0	7	7	3	3
38	*ybaZ*	4	0	2	1	0	0	7	7	3	3
39	*ycfJ*	5	0	2	0	0	1	8	7	3	2
40	*ydcW*	5	0	2	0	0	1	8	7	3	2
41	*ydjB*	4	0	2	0	1	0	7	7	3	3
42	*yebC*	5	0	2	0	0	2	9	7	3	2
43	*yedF*	5	0	0	0	2	2	9	7	3	2
44	*ydfT*	5	0	0	1	0	0	6	6	2	2
45	*cchB*	4	1	1	0	0	0	6	6	3	3
46	*chaA*	2	3	1	0	0	0	6	6	3	3
47	*cyaA*	1	3	2	0	0	0	6	6	3	3
48	*eutL*	3	1	2	0	0	0	6	6	3	3
49	*fadR*	2	0	2	0	2	0	6	6	3	3
50	*hybF*	3	1	2	0	0	0	6	6	3	3
51	*ilvL*	5	0	1	0	0	0	6	6	2	2
52	*napG*	4	0	2	0	0	0	6	6	2	2
53	*narH*	5	0	1	0	0	0	6	6	2	2
54	*nuoG*	5	0	1	0	0	0	6	6	2	2
55	*otsB*	2	0	2	1	1	0	6	6	4	4
56	*purR*	5	0	1	0	0	0	6	6	2	2
57	*rseA*	5	0	1	0	0	1	7	6	3	2
58	*trxA*	2	3	0	0	1	0	6	6	3	3
59	*wzzB*	3	0	1	0	2	0	6	6	3	3
60	*yaaX*	3	1	2	0	0	0	6	6	3	3
61	*ybhA*	5	0	1	0	0	0	6	6	2	2
62	*yddE*	4	0	2	0	0	0	6	6	2	2
63	*yeiH*	4	0	2	0	0	1	7	6	3	2
64	*yfgL*	3	0	2	0	1	0	6	6	3	3
65	*yjgA*	5	0	1	0	0	1	7	6	3	2
66	*yqjH*	5	0	1	0	0	0	6	6	2	2
67	*znuB*	5	0	0	0	1	0	6	6	2	2
68	*argK*	4	0	1	0	0	0	5	5	2	2
69	*aroD*	0	2	1	2	0	0	5	5	3	3
70	*astC*	3	0	1	0	1	2	7	5	4	3
71	*citE*	2	1	2	0	0	1	6	5	4	3
72	*hrpA*	1	3	1	0	0	0	5	5	3	3
73	*hycA*	4	0	1	0	0	0	5	5	2	2
74	*hycF*	4	0	1	0	0	0	5	5	2	2
75	*hycG*	3	0	2	0	0	0	5	5	2	2
76	*hyfI*	4	0	1	0	0	1	6	5	3	2
77	*narK*	4	0	1	0	0	1	6	5	3	2
78	*narY*	2	0	2	1	0	0	5	5	3	3
79	*nuoN*	4	0	1	0	0	0	5	5	2	2
80	*oppA*	3	0	2	0	0	0	5	5	2	2
81	*paaH*	3	0	2	0	0	1	6	5	3	2
82	*psiF*	3	1	1	0	0	0	5	5	3	3
83	*rne*	0	3	2	0	0	0	5	5	2	2
84	*soxS*	2	1	2	0	0	0	5	5	3	3
85	*tig*	5	0	0	0	0	0	5	5	1	1
86	*trg*	2	1	1	1	0	0	5	5	4	4
87	*tyrP*	4	0	0	1	0	0	5	5	2	2
88	*usg*	5	0	0	0	0	1	6	5	2	1
89	*yagF*	5	0	0	0	0	0	5	5	1	1
90	*yajI*	3	1	1	0	0	1	6	5	4	3
91	*ybfF*	4	0	1	0	0	1	6	5	3	2
92	*ybiB*	4	0	1	0	0	0	5	5	2	2
93	*ycbC*	0	3	2	0	0	2	7	5	3	2
94	*ycdZ*	4	0	1	0	0	0	5	5	2	2
95	*ycfD*	4	0	1	0	0	1	6	5	3	2
96	*ydhF*	4	0	0	1	0	0	5	5	2	2
97	*ydhP*	2	0	1	0	2	0	5	5	3	3
98	*yebS*	3	0	2	0	0	1	6	5	3	2
99	*yebZ*	3	0	2	0	0	1	6	5	3	2
100	*yegW*	3	0	2	0	0	0	5	5	2	2

**Figure 4:**
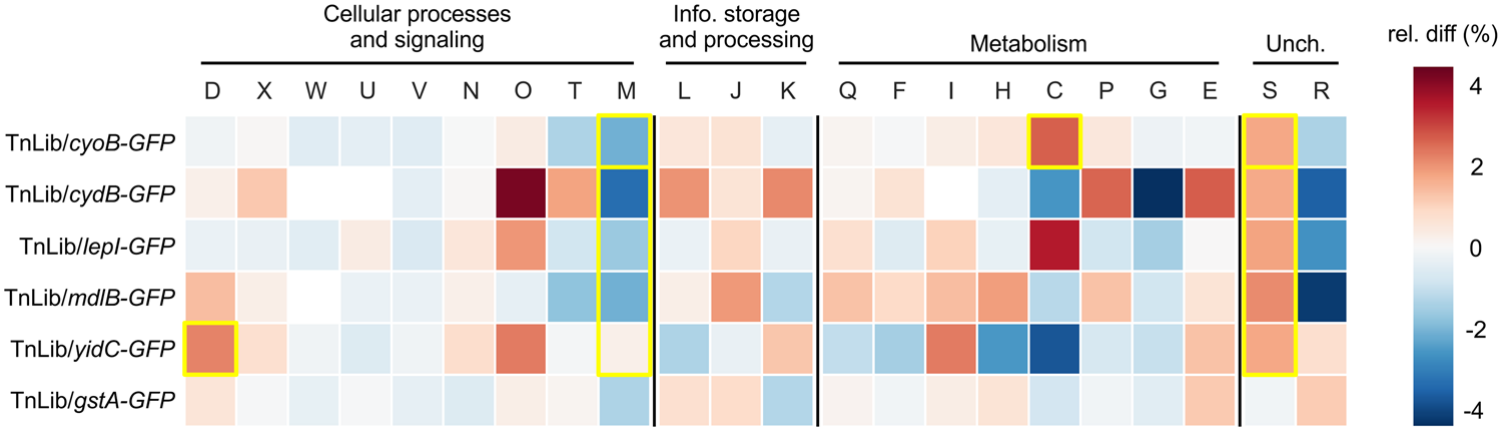
Changes in protein functional group frequency of enriched candidates relative to WT *E*. *coli* genome. The frequency of broad protein functional groups, COGs (full descriptions in Table S3), for enriched gene disruptions was compared to that of the WT *E*. *coli* genome, and instances passing Fisher’s exact test (p ≤ 0.05) are outlined in yellow. The library expressing a soluble protein (TnLib/*gstA-GFP*) shows very little change relative to WT. Group D (cell division) increased relative to the WT genome for TnLib/*yidC-GFP* (Fisher’s exact test, p = 0.023). In TnLib/*cyoB-GFP*, group M (cell membrane biogenesis) decreased and groups C (energy production and conservation) and S (unknown function) increased relative to the WT genome (Fisher’s exact test, p = 0.024, 0.020, and 0.048, respectively). Across all TnLib/IMP, group M (membrane biogenesis) decreased and group S (unknown function) increased relative to the WT genome (Fisher’s exact test, p = 0.014 and 0.014, respectively).

To determine if a particular protein class was consistently altered in all enriched TnLib/IMP candidates, we determined the distribution of broad protein functional groups (cluster of orthogonal groups, COGs, full description in Table S3)^36^. The difference in frequency of each COG was calculated between TnLib/IMP enriched gene disruptions and the WT *E*. *coli* genome (Fig. 4). Comparisons passing a Fisher’s exact test (p < 0.05) are highlighted with yellow boxes. The positive enrichment of a COG represents protein categories detrimental to IMP-GFP expression (Fig. 4, blue), and depleted abundance represents groups potentially beneficial to IMP-GFP expression (Fig. 4, red). Very little change in COG frequency was evident for the library expressing the soluble protein (Fig. 4, TnLib/*gstA-GFP*). Conversely, the libraries expressing membrane proteins display some consistent trends. The COG representing proteins with unknown function increased in frequency relative to WT (group S, Fisher’s exact p = 0.014), while that for cell membrane biogenesis proteins decreased in frequency (group M, Fisher’s exact p = 0.014). The low enrichment of cell membrane biogenesis candidates suggests that these proteins are beneficial to IMP expression. From the metabolism category, energy production and conservation proteins showed the greatest divergence from the *E*. *coli* genome, with TnLib/*cyoB-GFP* exhibiting a significant decrease (group C, Fisher’s exact p = 0.020). This supports previous work showing that central metabolism is altered in strains expressing IMPs (Fig. 4)^1^. In addition, cell division proteins showed a strong increase in TnLib/*yidC-GFP* (group D, Fisher’s exact p = 0.023). Generally, the frequency of most protein functional groups for enriched gene disruptions was not similar across all TnLib/IMP tested.

### Validation of Methodology in Single-Gene Deletion Strains

**Figure 5:**
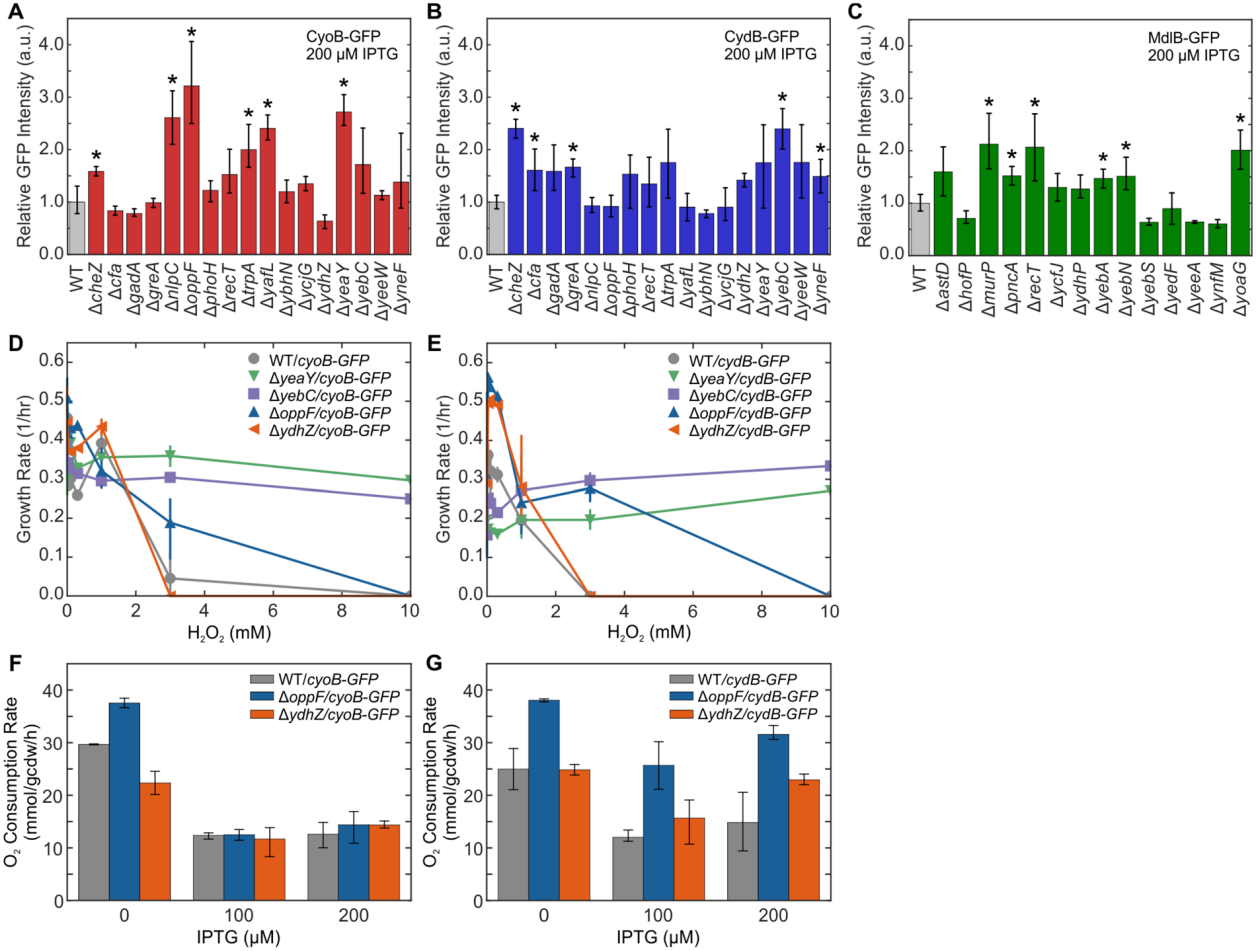
Single gene deletions of corresponding candidate transposon mutants increased membrane protein expression and function. (**A**–**C**) Mean GFP intensity of single gene deletion backgrounds was compared to WT *E*. *coli* (n = 5 biological replicates, error bars represent SEM, asterisks indicate two-tailed t-test p < 0.05). The greatest increase observed was with (A) *ΔoppF* increasing Cyo expression 3-fold and (**B**) *ΔcheZ* and *ΔyebC* increasing Cyd expression 2.5-fold. (**D**,**E**) To evaluate functional expression of the Cyo and Cyd complexes, hydrogen peroxide sensitivity was measured for WT, *ΔyeaY*, *ΔyebC*, *ΔoppF*, and *ΔydhZ* expressing the Cyo or Cyd complexes (n ≥ 3, error bars represent SEM). Deletion strains *ΔyeaY* (green upside down triangles), *ΔyebC* (purple squares), and *ΔoppF* (blue triangles) demonstrated increased resistance to hydrogen peroxide when expressing either the Cyo or Cyd complex relative to WT (grey circles). (F-E) Dissolved oxygen was measured for WT, *ΔoppF*, and *ΔydhZ* expressing the Cyo or Cyd complexes (n ≥ 3, error bars represent SEM). The oxygen consumption rate was the same across all strains when expressing the Cyo complex whereas the oxygen consumption rate of *ΔoppF/cydB-GFP* increased relative to WT/*cydB-GFP*. See also Fig. S5.

To validate enriched gene disruptions, we interrogated 30 corresponding single-gene deletion strains with variable enrichment ratios in *E*. *coli* BW25113 from the Keio Collection^37^ for the ability to express a desired IMP. Of the seventeen candidates tested for CyoB-GFP and CydB-GFP expression, 12 were enriched in TnLib/*cyoB-GFP* and 4 were enriched in TnLib/*cydB-GFP*; of the fourteen candidates tested for MdlB-GFP expression, 3 were enriched in TnLib/*mdlB-GFP* (Table S4). The IMP-GFP signal was measured by flow cytometry for at least 5 biological replicates induced with 200 µM IPTG after 24 hours. Mean IMP-GFP signal of the deletion strains was compared to WT *E*. *coli* expression of CyoB-GFP, CydB-GFP, and MdlB-GFP (Fig. 5A–C). The mean IMP-GFP signal for WT expression was highly variable (Fig. S5a), thus for clarity error bars represent the confidence interval of the knockout IMP-GFP.

Seventeen candidates were chosen from TnLib/*cyo-GFP* and TnLib/*cyd-GFP*. Of those seventeen, 6 and 5 knockouts significantly increased the CyoB-GFP and Cyd-GFP, respectively (Fig. 5A,B, asterisks, two-tailed t-test, p < 0.05, Table S4). The largest increase in mean IMP-GFP relative to WT occurred with ∆*oppF*, increasing CyoB-GFP signal 3-fold. An additional 4 mutants (∆*nlpC*, ∆*trpA*, ∆*yafL*, and ∆*yeaY*) increased CyoB-GFP signal more than 2-fold over WT expression. Only two mutants that showed significant change with CydB-GFP (∆*cheZ* and ∆*yebZ*) increased mean CydB-GFP signal more than 2-fold over WT expression (Fig. 5B).

Five candidates tested from TnLib/*mdlB-GFP* showed a statistical increase of mean MdlB-GFP signal over WT expression (Fig. 5C, asterisks, two-tailed t-test, p < 0.05, Table S4), but the magnitude of this increase was relatively low compared to hits found for CyoB-GFP and CydB-GFP. Only two mutants (*∆murP* and ∆*yoaG*) increased the mean MdlB-GFP signal 2-fold over WT expression (Fig. 5C). In addition, WT/*mdlB-GFP* exhibited a large variation in MdlB-GFP signal. A noteworthy nuance is that some deletion strains (∆*yebA*, ∆*ynfM*, ∆*yeeA*, and ∆*yebS*) markedly increased homogeneity of the population with IMP-GFP signal, but only ∆*yebA* simultaneously increased mean IMP-GFP signal intensity (Fig. S5a).

We also determined if increased expression of the Cyo or Cyd complexes in single-gene deletion strains confer enhanced functionality of these respiration complexes. An improved function requires correctly folded and localized protein complexes providing a validation beyond fluorescence measurement. We carried out two analogous functional assays for the expression of the Cyo and Cyd complexes: hydrogen peroxide sensitivity and oxygen consumption rate (Fig. 5). Strains deleted for the entire *cyoABCD* or *cydAB* operon exhibit sensitivity to exogenous hydrogen peroxide^38,39^. We hypothesized that increased expression of functional Cyo or Cyd complexes would result in strains that were more resistant to hydrogen peroxide; thus WT *E*. *coli* and single deletion strains ∆*oppF*, ∆*yeaY*, ∆*yebC*, and ∆*ydhZ* were tested for the ability to grow when subjected to exogenous hydrogen peroxide. The ∆*ydhZ* strain serves as a control for a knockout that does not increase CyoB-GFP or CydB-GFP signal. All base strains showed similar peroxide toxicity (Fig. S5), and uninduced strains exhibited similar growth rates in the absence of H_2_O_2_ (Fig. 5D,E). The deletion strain with no change in IMP expression, ∆*ydhZ* (Fig. 5A,B), showed similar sensitivity to H_2_O_2_ as WT cells. Consistent with the 2-fold increase in IMP-GFP signal, expression of Cyo and Cyd complexes in *∆yeaY* and ∆*yebC* resulted in higher growth rates when exposed to H_2_O_2_ when compared to WT (Fig. 5D,E). Interestingly, ∆*oppF* showed the greatest increase in CyoB-GFP signal (Fig. 5D), but ∆*oppF/cyoB-GFP* cells did not confer resistance to H_2_O_2_. In contrast, ∆*oppF* did not increase in CydB-GFP signal, but we observed an increase in H_2_O_2_ resistance compared to WT in ∆*oppF/cydB-GFP* at concentrations up to 3 mM H_2_O_2_.

Additionally, we measured dissolved oxygen consumption of WT, ∆*oppF* and ∆*ydhZ* in a sealed microtiter plate as an orthogonal test for functional expression of the Cyo and Cyd complexes. Dissolved oxygen (DO) normalized to cell density (OD_600nm_) showed that ∆*oppF/cydB-GFP* consumed dissolved oxygen faster than WT/*cydB-GFP* and ∆*ydhZ/cydB-GFP* (Fig. S5). In the absence of IPTG, WT and ∆*ydhZ* exhibited similar oxygen consumption rates (~25 mmol/gcdw/h) while ∆*oppF* exhibited a higher baseline oxygen consumption rate (~38 mmol/gcdw/h, Fig. 5F,G). Upon induction, WT/*cyoB-GFP* and WT/*cydB-GFP* showed a two-fold decrease in oxygen consumption rate (~13 mmol/gcdw/h) consistent with detrimental impact of IMP expression on growth (Fig. 5F,G). Both ∆*oppF/cyoB-GFP* and ∆*ydhZ/cyoB-GFP* oxygen consumption rates were similar to WT/*cyoB-GFP* (Fig. 5F). However, ∆*oppF/cydB-GFP* induced with 100 µM IPTG increased oxygen consumption rate (25.7 ± 5.1 mmol/gcdw/h) compared to WT/*cydB-GFP* (12.0 ± 1.4 mmol/gcdw/h) (Fig. 5G, two-tailed t-test, p = 0.007). The inconsistency between expression and function emphasize that the cellular burden of IMP overexpression on the cell is complex and idiosyncratic, likely due to the localization, maturation, and function of these proteins. Overall, we found that it is possible to obtain host chasses that improve a desirable phenotype using this genome-wide search for membrane protein expression determinants.

## Discussion

Membrane proteins are vital to the viability and metabolism of a cell, typically comprising 20% of genes in microbial genomes^40,41^. Despite this significance, only 4.4% of bacterial crystal structures deposited in the Protein Databank (PDB) currently are transmembrane proteins, pointing to their challenging nature to overexpress (Supplemental Text). Similar limitations exist in the use of the membrane proteins in strain engineering. Among other strategies to improve microbial membrane protein expression, decreasing RNA transcription partially alleviates IMP expression toxicity^4,17^. However, transcriptional burden is only partially responsible for recombinant membrane protein toxicity. Several strains designed for membrane protein expression, such as Lemo21/pLemo, SuptoxR, and SuptoxS, restrict co-expression plasmid options due to required internal plasmid systems. Our approach identifies genomic alterations that improve membrane protein expression, making these edits potentially tractable for scale-up bioproduction applications.

Our study illustrates a generalizable method that leverages next generation sequencing in tandem with FACS to discover gene targets that negatively impact IMP expression in *E*. *coli*. The fluorescence intensity of the IMP-GFP fusion was used to assess IMP expression levels. The use of GFP fusions as a proxy for protein expression is an established approach but should be considered a starting point beyond which the protein of interest needs to be evaluated directly using functional assays. Our approach identified gene disruptions that accommodate increased expression of an IMP without a penalty in growth. FACS restrains the throughput and number of cells (typically 1 × 10^6^ cells per sorted sample) for amplification and sequencing. The enrichment ratios used to track the sorted gate at which gene disruptions appeared in enriched abundance potentially include ~25% false positives, but collecting more replicates counters this uncertainty.

The gene disruptions enriched in TnLib/IMP suggest that membrane protein complexes pose an idiosyncratic burden on the cell. Of the 14 common candidates were enriched in four out of the five libraries tested (Fig. 2D, Table 2), one is *tatA*, a component of the twin arginine translocation (Tat) complex. None of the IMPs examined in this study use the Tat translocation machinery, and the disruption of *tatA* would be potentially disruptive to the export of fully folded proteins^42,43^. The frequency of metabolism proteins in the enriched High GFP population varied from that of WT *E*. *coli*, but this response was not consistent across all IMPs tested (Fig. 4). Though several candidates tested in the validation improved IMP expression, we did not find a mutant that universally increased expression of the IMP-GFPs tested (Fig. 5A–C). No single mechanism universally or equally improved membrane protein production across all IMPs, pointing to the complexity of IMP expression stress response. However, as more proteins get tested across a range of bacterial backgrounds, it may become possible to categorize membrane proteins into groups that have a similar suite of loci that can be edited for their expression.

There are examples in the literature investigating genomic alterations that improve membrane protein expression. For instance, the Walker strains, C41(DE3) and C43(DE3), were isolated from selective pressure of plasmid-based IMP expression^15^. The genomic mutations and deletions in C41(DE3) and C43(DE3) were recently described in detail^16,17^. Two large deletion regions were found in the genome of C43(DE3): *yjiV*–*lgoD* and *ccmF*–*ompC* ^16^. We examined the enrichment ratios of genes from this region to determine if our study identified genes as candidates for increased IMP expression. While we observed few significant candidates in the *yjiV*–*lgoD* C43(DE3) deletion region, several gene disruptions from the *ccmF*–*ompC* C43(DE3) deletion region were enriched (Fig. 3, *ccmF*–*ompC*). Specifically, gene disrutions in *ccmAB*, *napBCGH*, *mgo*, *ada*, and *ftp* were enriched in TnLib/*cyoB-GFP*, TnLib/*lepI-GFP*, and TnLib/*yidC-GFP*. This general enrichment indicates that these genes are detrimental to the expression of IMPs. This similarity between deletions in C43(DE3) and disrupted gene enrichment show that our strategy identified authentic and physiologically relevant genetic deletions that are beneficial to IMP expression. It is noteworthy however that, overexpression of our target IMP complexes in deletion strains identified in this study outperformed the same overexpression constructs in the DE3 Walker strain (Fig. S6) further supporting the need for IMP specific host genome engineering. Several candidates identified from the enrichment ratio (R_High/Low_) of TnLib/*cyoB-GFP* and TnLib/*cydB-GFP* increased IMP-GFP signal and function (Fig. 5). Fifteen out of 30 deletion mutants screened in validation increased IMP-GFP signal, 10 of which increased IMP-GFP signal by 2- to 3-fold over the corresponding WT IMP-GFP expression (Fig. 5A–C).

The strength of our approach is capturing the enrichment of gene disruptions that decrease the cellular burden of IMP expression. We identified *E*. *coli* mutant strains capable of increased ability to express an IMP with reduced growth impact. While single locus disruptions can enable the over-expression of a membrane protein, our results emphasized that there are many constraints which dictate membrane protein expression, and a universal locus that is equally effective for all membrane protein expression needs is unlikely. Our strategy provides a generalizable method that may be used to optimize a host microbe for the expression of any toxic protein, protein complex or high-burden pathway, thereby improving the complex synergy between the viability of the microbial host and the expression of a protein or pathway.

## Materials and Methods

### Strains

The *E*. *coli* BW25113 transposon insertion library^27^ was obtained from the Deutschbauer lab (amdeutschbauer@lbl.gov). Strains and plasmids are listed in Table S5. *E*. *coli* BW25113 was used as the wild-type strain for the construction of the transposon library and control strains (Table 1). Cells were grown routinely grown in EZ Rich Medium supplemented with 1% glucose, 50 µg/mL kanamycin (library antibiotic resistance), and 30 µg/mL chloramphenicol (plasmid antibiotic resistance) at 30 °C. Single gene deletion strains^37^ were used for testing the candidate mutants from the transposon library. Cloning was performed in *E*. *coli* DH10B. Strains are also listed in the JBEI Public Registry (public-registry.jbei.org/folders/336).

### Plasmid design and assembly

All plasmids were constructed using Golden Gate cloning into the Bgl brick plasmid pBbA5c^44^. All IMPs in this study are known to have C-termini that are facing the cytoplasm. IMP genes of interest (Table 1) were amplified from MG1665 genomic DNA using the primers designed by J5^45^ in Table S5 with PrimeStar DNA polymerase. Superfolding GFP (GFP) with a 14 aminoacid TEV linker (S V P G S E N L Y F Q G Q F) was designed based on an established library of IMP-GFP fusion proteins^41,46^, was ordered as a gBlock from IDT. Finally, TEV-GFP was cloned into the C-terminus of the IMP on pBbA5c. Plasmids were sequence verified and stored as glycerol stocks at −80 °C in *E*. *coli* DH10B. All plasmids are listed in Table S5 and plasmid sequences are available from the JBEI Public repository (public-registry.jbei.org/folders/336). All resources used are listed in the Supplementary note.

### Transformation of plasmid DNA into BW25113 pooled transposon library (TnLib)

The BW25113 *E*. *coli* transposon library developed by Wetmore and coworkers was used to find genetic mutations that increase membrane protein expression^27^. To make the TnLib electrocompetent, the pooled library was grown to 0.5 OD_600nm_ in 1.5 L LB supplemented with 50 μg/mL kanamycin at 37 °C. The culture was chilled on ice and washed twice in ice-cold water and twice in ice-cold 10% glycerol. The cells were resuspended in 10% glycerol, flash frozen, and stored at −80 °C. Electrocompetent TnLib was transformed with 10 ng plasmid DNA with 2500 V in 2 mm electroporation cuvettes. At least 4 electroporation samples were pooled to ensure full coverage of the bar codes. The transformed cells were recovered at 37 °C in SOC medium for 1 h. Cells containing plasmid DNA were selected for by diluting to 0.1 OD_600nm_ in LB supplemented with 50 μg/mL kanamycin and 30 μg/mL chloramphenicol and outgrowth at 37 °C. Once cultures reached 1.0 OD_600nm_, the cultures were pelleted and resuspended to 10.0 OD_600nm_ in LB supplemented with 10% glycerol. Single use aliquots were flash frozen and stored at −80 °C. An aliquot of the transformed TnLib was used to confirm that the liquid selection resulted in at least 99.5% cells harboring the candidate IMP-GFP plasmid DNA. The genomic DNA was purified using the Qiagen DNeasy Blood and Tissue Kit, and the diversity of all libraries was confirmed using BarSeq^27^. All resources used are listed in the Supplementary note.

### Growth and induction of IMP-GFP in TnLib

An aliquot of the TnLib harboring a particular plasmid was thawed at room temperature and diluted to 0.1 OD_600nm_ in EZ rich supplemented with 1% glucose, 50 μg/mL kanamycin, 30 μg/mL chloramphenicol, and variable IPTG concentration. Growth and whole culture GFP was evaluated in 48-well plates at 30 °C for 24 h using a Tecan F200 plate reader (Tecan, Männedorf, Switzerland). A secondary growth phase occurred in all TnLib/IMP grown under high induction concentrations due to plasmid loss (Fig. S1). This was only observed with TnLib/IMP treated with high inducer concentrations and at later time points (≥10 h). To minimize plasmid-loss bias, we halted growth at late exponential phase (6 h) for all subsequent sorting experiments. All resources used are listed in the Supplementary note.

### Identification of enriched candidates from the Transposon Library

Cells expressing a particular IMP-GFP fusion were grown until exponential phase, 6 h, as described above, immediately chilled on ice, and analyzed by flow cytometry. Specifically, a 1–5 µL aliquot of each culture was diluted into 0.5 mL of 0.22-µm sterile-filtered phosphate buffered saline (pH 7.4) and loaded into the flow cytometer. At least 10,000 events were captured for each sample using a FACS Aria II Flow Cytometer and Cell Sorter (BD Biosciences, San Jose, CA). Sorting gates were drawn in forward (FSC-A) and side (SSC-A) scatter to exclude non-cellular debris and electronic noise based on a 0.22-μm filter sterilized phosphate buffered saline control. Secondary gates were drawn for the isolation of No, Low, and High GFP on the GFP (FITC-A) histogram. For each gate, 1 × 10^6^ cells were isolated. The genomic DNA (gDNA) from each gate was purified using the Qiagen DNeasy Blood and Tissue Kit. The bar codes from all loci disruptions were amplified using BarSeq PCR (primers; Table S5). Specifically, bar codes were amplified from gDNA using Q5 DNA polymerase with 0.5x Q5 GC enhancer and the standard Q5 reaction buffer under the following cycling conditions: 98 °C for 4 min followed by 30 cycles of 30 s at 98 °C, 30 s at 55 °C, and 30 s at 72 °C, followed by a final extension at 72 °C for 5 min. Amplification was verified by electrophoresis, and the ~200 bp product was purified with the Zymo Clean & Concentrator kit. The dsDNA concentration of each PCR reaction was measured by Qubit dsDNA HS assay kit. Equal masses of up to 14 samples with unique Illumina adapters were multiplexed and sequenced by MiSeq using a MiSeq Reagent Kit v2 50-cycle kit. Relative bar code abundance (log_2_ fitness) was calculated as previously described using FEBA (https://bitbucket.org/berkeleylab/feba)^27^. Log_2_ fitness values are listed in Table S1. Comparisons of log_2_ fitness values were used to determine which bar-coded strains were in high abundance in the Low GFP and High GFP gates compared to the No GFP gates, resulting in R_High/No_ and R_Low/No_ as shown in Equations 1 and 2. Similarly, the High GFP and Low GFP gates were compared, resulting in R_High/Low_ as shown in Equation 3. These ratios are referred to as enrichment ratios.

1 $${R}_{High/No}=\,lo{g}_{2}{f}^{HighGFP}-lo{g}_{2}{f}^{NoGFP}$$

2 $${R}_{Low/No}=\,lo{g}_{2}{f}^{LowGFP}-lo{g}_{2}{f}^{NoGFP}$$

3 $${R}_{High/Low}=\,lo{g}_{2}{f}^{HighGFP}-lo{g}_{2}{f}^{LowGFP}$$

Enrichment ratio values are listed in Tables S1 and S2. Transposon insertion candidates with ratios above values of +2.0 are considered enhanced in the population. Circos^47^ was used to map enriched gene disruptions to genome location. All resources used are listed in the Supplementary note.

### Flow cytometry of single-gene deletion strains

Gene knockouts were selected to verify changes in membrane protein expression using the Keio collection^37^ and are listed in Table S5. Each strain grown from the Keio collection was verified by colony PCR. The strains in the Keio collection and the base strain, *E*. *coli* BW25113, were transformed with plasmid DNA encoding an IMP-GFP fusion. Cell culture populations were analyzed by flow cytometry using a BD Accuri C6 (BD Biosciences, San Jose, CA). Five colonies of each sample were grown in 600 µL EZ rich medium supplemented with 1% glucose, and 30 μg/mL chloramphenicol until 0.5 OD_600nm_ and induced for 24 h at 30 °C with 200 µM IPTG in 96-well deep-well plates. Samples were handled identically for the TnLib/*mdlB-GFP* except cultures were prepared in 5 mL volumes in glass culture tubes. For analysis on the BD Accuri Flow Cytometer, 2 μL of culture was diluted into 100 μL 0.22-μm filter sterilized phosphate buffered saline (pH 7.4), and 50,000 events were collected for every sample. Analysis gates were drawn to exclude non-cellular debris and electronic noise based on a 0.22-μm filter sterilized phosphate buffered saline control. Mean GFP signal of each knockout strain was compared to the mean GFP signal of WT *E*. *coli*. All resources used are listed in the Supplementary note.

### Hydrogen peroxide sensitivity assay

Single colonies were inoculated into 5 mL LB supplemented with 30 μg/mL chloramphenicol and grown overnight at 30 °C. From overnight cultures, 5 µL was diluted in 100 μL LB 1% glucose supplemented with 30 μg/mL chloramphenicol, variable IPTG concentration, and variable hydrogen peroxide concentration in a 96-well microtiter plates. Plates were covered with a semi-permeable USA Scientific Breathe-Easy film and assayed for growth in a BioTek Synergy (BioTek Instruments, Inc, Winooski, VT, USA) plate reader at 30 °C for the duration of the experiment. Gas impermeable films impact the lag time and sensitivity of wild-type strains to exogenous hydrogen peroxide. All resources used are listed in the Supplementary note.

### Bacterial oxygen consumption

Single colonies were inoculated into 5 mL EZ rich supplemented with 1% glucose and 30 μg/mL chloramphenicol, and cultures were grown overnight at 30 °C. From overnight cultures, 2 µL was diluted in 800 μL EZ rich supplemented with 30 μg/mL chloramphenicol and 0, 100, or 200 µM IPTG in an m2p labs Biolector FlowerPlate. Plates were sealed with an Axygen gas impermeable film to limit gas exchange. The plates were processed in a BioLector to measure biomass and dissolved oxygen. All resources used are listed in the Supplementary note.

### Growth Rate Analysis

Growth rate was calculated using a sliding window linear fit of the natural log of OD_600_ over time with a 3 h window. The linear fit with maximum growth rate with a Pearson correlation greater than 0.95 was reported. Averages of at least 5 biological replicates are shown in Fig. S1 with error bars representing 95% confidence intervals.

### Analysis of Enrichment Ratio False Positive Rate for Biological Replicates

Biological replicates of uninduced TnLib/IMP sorted in the No GFP gate were compared to determine the day-to-day variability of enrichment ratios. Enrichment ratios of these samples from separate MiSeq runs were compared (R_BioRep1_ and R_BioRep2_) using kernel density estimates. As no expression stress is applied in these samples, most gene interruptions were expected to have little change in log2 fitness and, likewise, an enrichment ratio close to 0. Kernel density estimate analysis shows that most gene disruption enrichment ratios lie at or near the origin (Fig. S4). To approximate a false positive rate for the enrichment ratio, the percent of data outside of the enriched threshold (±2.0) was determined. We interpret the percent of data outside ±2.0 range as the coefficient of variance for the enrichment ratio and report this percent as the false positive rate.

### COG Distributions

The percent of each cluster of orthogonal groups (COG) was assigned according to the available *E*. *coli* database^36^ (Table S3). The percent of each COG was calculated for each TnLib/IMP and compared to the percent of the corresponding COG in the total WT *E*. *coli* genome (Fig. 4). The significance of these changes relative to the WT *E*. *coli* genome were determined using a Fisher’s exact test, and COGs with Fisher’s exact p ≤ 0.05 were considered significantly different from the WT *E*. *coli* genome.

### Oxygen Consumption Rate Calculation

The oxygen consumption rate was calculated for 3 biological replicates as the growth rate multiplied by the rate of oxygen uptake per cell density (%DO/OD_600_/h). Units were converted to mmol/gcdw/h assuming that 100% DO is 0.21 mmol O_2_/L and 1.0 OD_600_ is equivalent to biomass of 0.4 gcdw/L. Error bars represent standard error of three biological replicates.

### Data

The raw BarSeq sequencing data and evaluated log_2_ fitness data have been deposited in the GEO database under ID code: GSE95857.

## Electronic supplementary material

Supplementary Figures S1-6 and Resource List

Table S1

Table S2

Table S3

Table S4

Table S5
