# Supplementary material for: Improving membrane protein expression and function using genomic edits
Source: Sci Rep. 2017 Oct 12;7:13030. doi: 10.1038/s41598-017-12901-7 (PMC5638813; doi:10.1038/s41598-017-12901-7)
Supplement: Supplementary file 1 — Supplementary Figures S1-6 and Resource List [file 41598_2017_12901_MOESM1_ESM.pdf]

Supplemental Figures and information

Improving membrane protein expression and function using genomic edits

Heather M. Jensen[1,2,], Thomas Eng[1,2,], Victor Chubukov[1,2,], Robin A. Herbert[1,2], and Aindrila Mukhopadhyay*[1,2]

[1]Joint BioEnergy Institute, Emeryville, CA 94608, USA.

[2]Biological Systems and Engineering Division, Lawrence Berkeley National Laboratory, Berkeley, CA 94720, USA.

Supplemental Figures.

Figure S1. Membrane protein expression in the transposon library results in decreased growth rate and GFP signal beyond toxic inducer concentration.

Figure S2. Toxic induction of IMP-GFP results in biomodal distribution of cells with and without GFP signal.

Figure S3. Related to Figure 2 and Table S1. Overview of Bar-seq results from FACS.

Figure S4. Related to Table S1. Reproducibility of biological replicate enrichment ratios.

Figure S5. Related to Figure 5 and Table S4. Supplemental data to single gene deletion validation experiments.

Figure S6. Comparison of the C43(DE3) Walker strain with deletion strains discovered in this study.

Supplemental Tables.

Table S1. All $\log_2$ fitness and enrichment ratios.

Table S2. Enriched gene deletion candidates.

Table S3. COG descriptions.

Table S4. Validation data metrics.

Table S5. Primers, plasmids, strains used in this study.

Supplemental Text.

Calculation of percent membrane protein entries in the Protein Databank (PBD), to-date.

Resource List

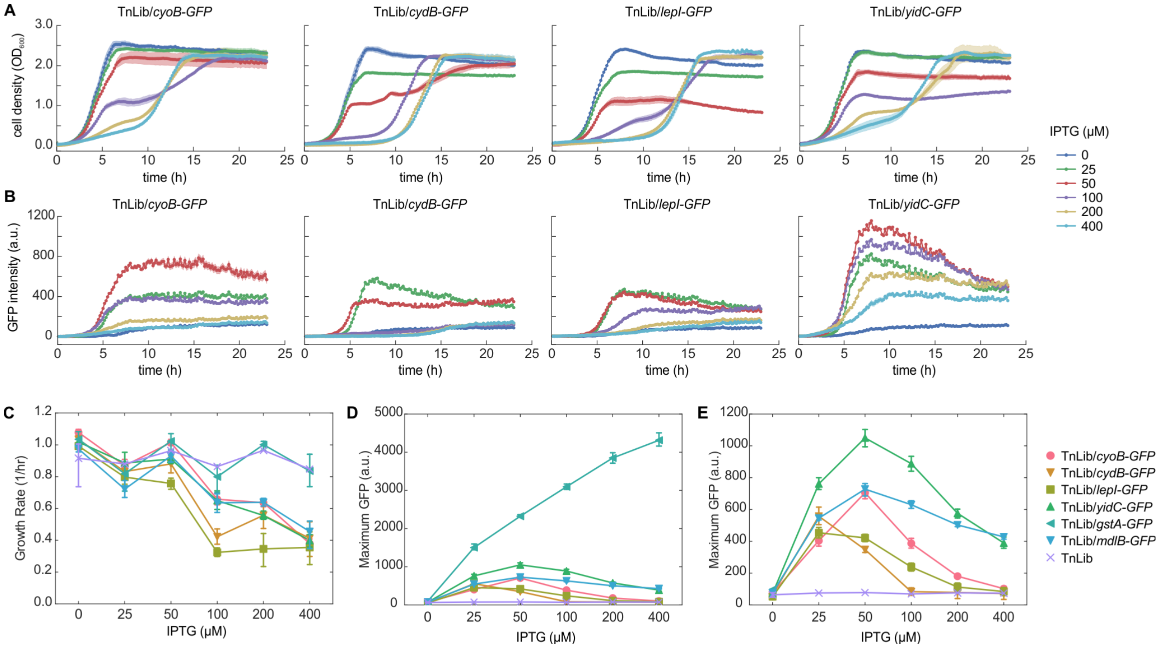

Figure S1. Membrane protein expression in the transposon library results in decreased growth rate and GFP signal beyond toxic inducer concentration. (A) Example growth curves of TnLib/IMP showed a decrease in growth rate and increase in growth lag with increasing inducer (IPTG) concentration. A secondary growth curve was observed in strains induced with high IPTG concentration after ~10 h, which is attributed to plasmid loss. All sorting experiments were carried out with strains grown for 6 h. (B) GFP signal intensity over time shows the IMP-GFP signal intensity in bulk culture. The IMP-GFP signal is greatest when the cell does not experience any deleterious growth impact. (C) Growth rate of TnLib/IMP decreases with increasing IPTG concentration. TnLib and TnLib/*gstA-GFP* show no toxicity to the addition of IPTG or expression of soluble protein. (D) Maximum GFP of soluble GST-GFP demonstrates a typical protein induction profile from non-toxic protein expression. (E) For all TnLib/IMP, maximum GFP decreases at toxic induction strengths. For panels C-D, data represents the mean of at least 5 biological replicates, and error bars represent 95% confidence intervals.

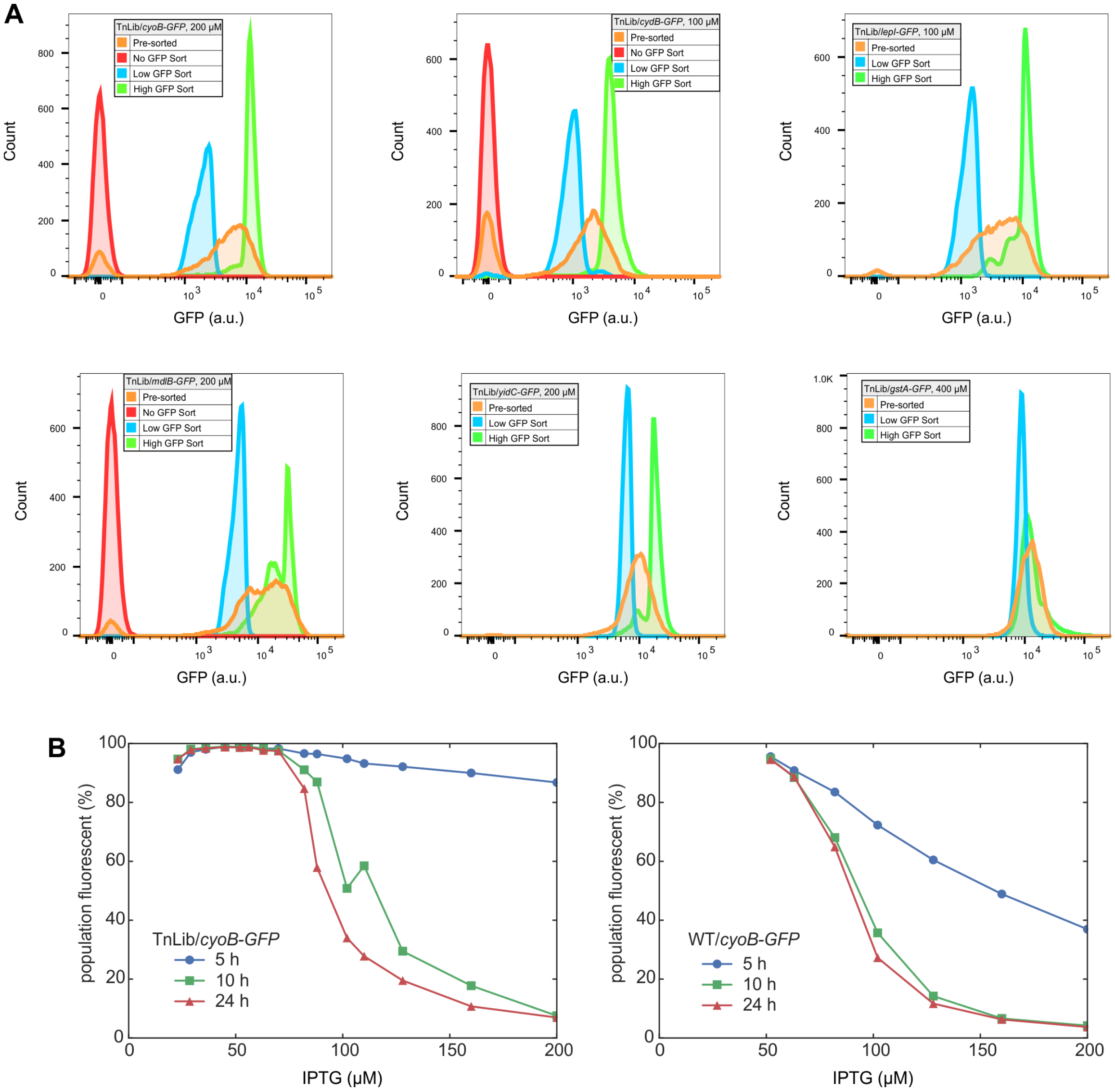

Figure S2. Toxic induction of IMP-GFP results in biomodal distribution of cells with and without GFP signal. (A) All TnLib/IMP studied exhibit a bimodal distribution of cells with and without GFP signal if induced past a critical concentration of IPTG. These examples demonstrate this bimodal distribution of the pre-sorted cells for TnLib/*cyoB-GFP*, TnLib/*cyd-GFP*, and TnLib/*mdlB-GFP* and the shown [IPTG] (orange lines). Below this critical inducer concentration, IMP-GFP signal is titratable and monodisperse, for example TnLib/*lepI-GFP* and TnLib/*yidC-GFP* at the shown [IPTG] (orange lines). TnLib/*gstA-GFP* did not ever display a bimodal distribution at the concentrations of IPTG tested. The remaining lines demonstrate sorting purity. Red lines represent cells sorted with No GFP signal. Blue lines represent cells sorted with Low GFP signal. Green lines represent cells sorted with High GFP signal. For each gate, one million cells were sorted, and the genomic bar codes were amplified for analysis with BarSeq. (B) The population of cells with GFP signal decreases as inducer concentration (IPTG) increases in TnLib/*cyoB-GFP* and WT/*cyoB-GFP*. Additionally, this spread of populations without GFP signal increased as the expression time increased from 5 h (blue circles) to 10 h (red triangles). These trends are consistent with the consequences of membrane protein expression observed in the literature.

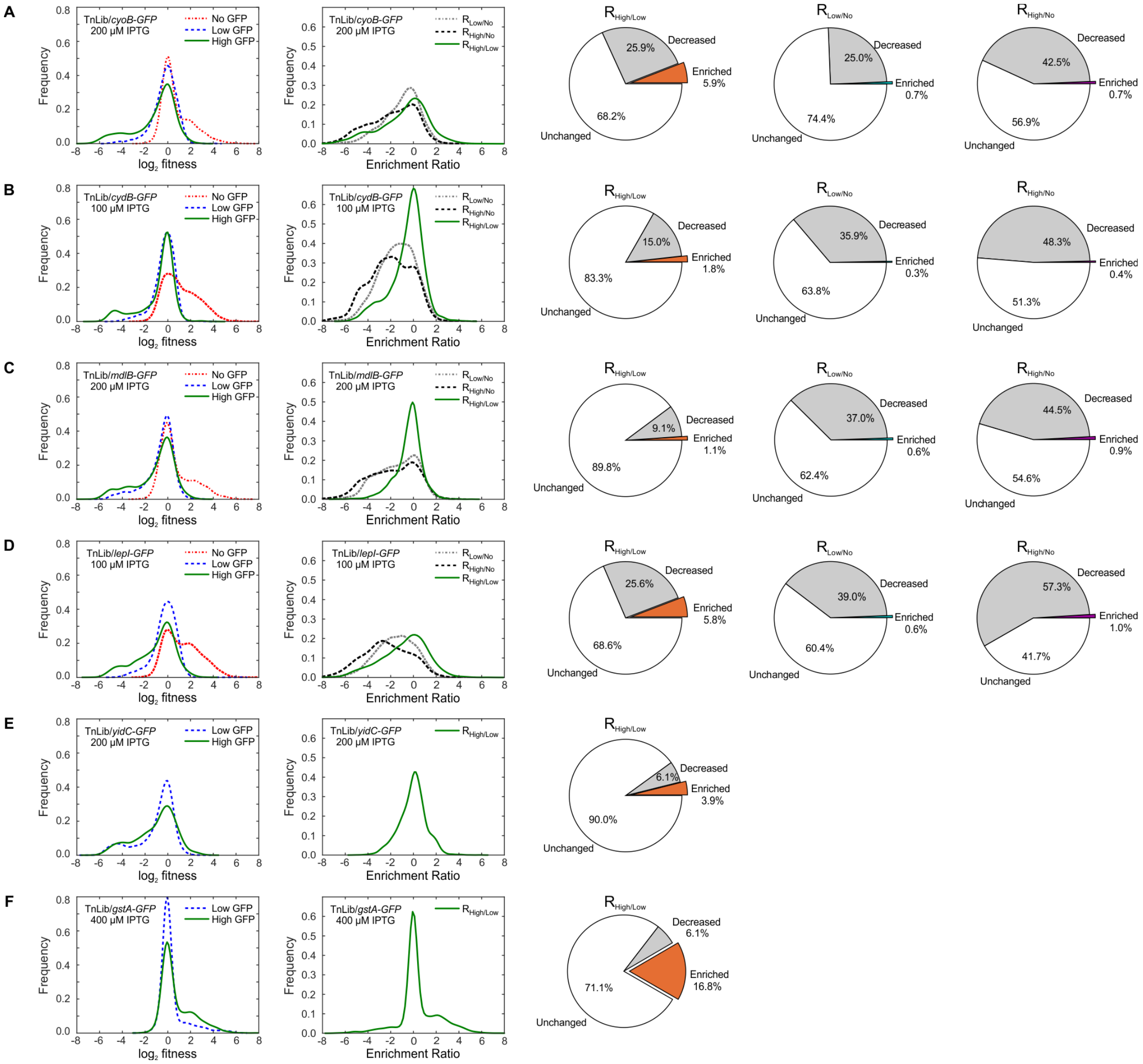

Figure S3. Related to Figure 2 and Table S1. Overview of Bar-seq results from FACS. The relative fitness of each bar-coded gene disruption strain was determined by BarSeq for sorted samples of (A) TnLib/*cyoB-GFP*, (B) TnLib/*cydB-GFP*, (C) TnLib/*mdlB-GFP*, (D) TnLib/*lepI-GFP*, E) TnLib/*yidC-GFP*, and soluble protein control TnLib/*gstA-GFP* (Left Panel). Comparison of gene disruption fitness between sorted gates result in enrichment ratios (Second Panel). Enrichment ratios above +2.0 and below -2.0 are considered enriched abundance or decreased abundance, respectively. For $R_{High/Low}$, 1 - 6% of barcoded strains are enriched (Center Panel, orange wedge). For $R_{High/No}$ and $R_{Low/No}$, only ~1% of bar-coded strains are enriched (Right Panels, cyan wedge and purple wedge, respectively).

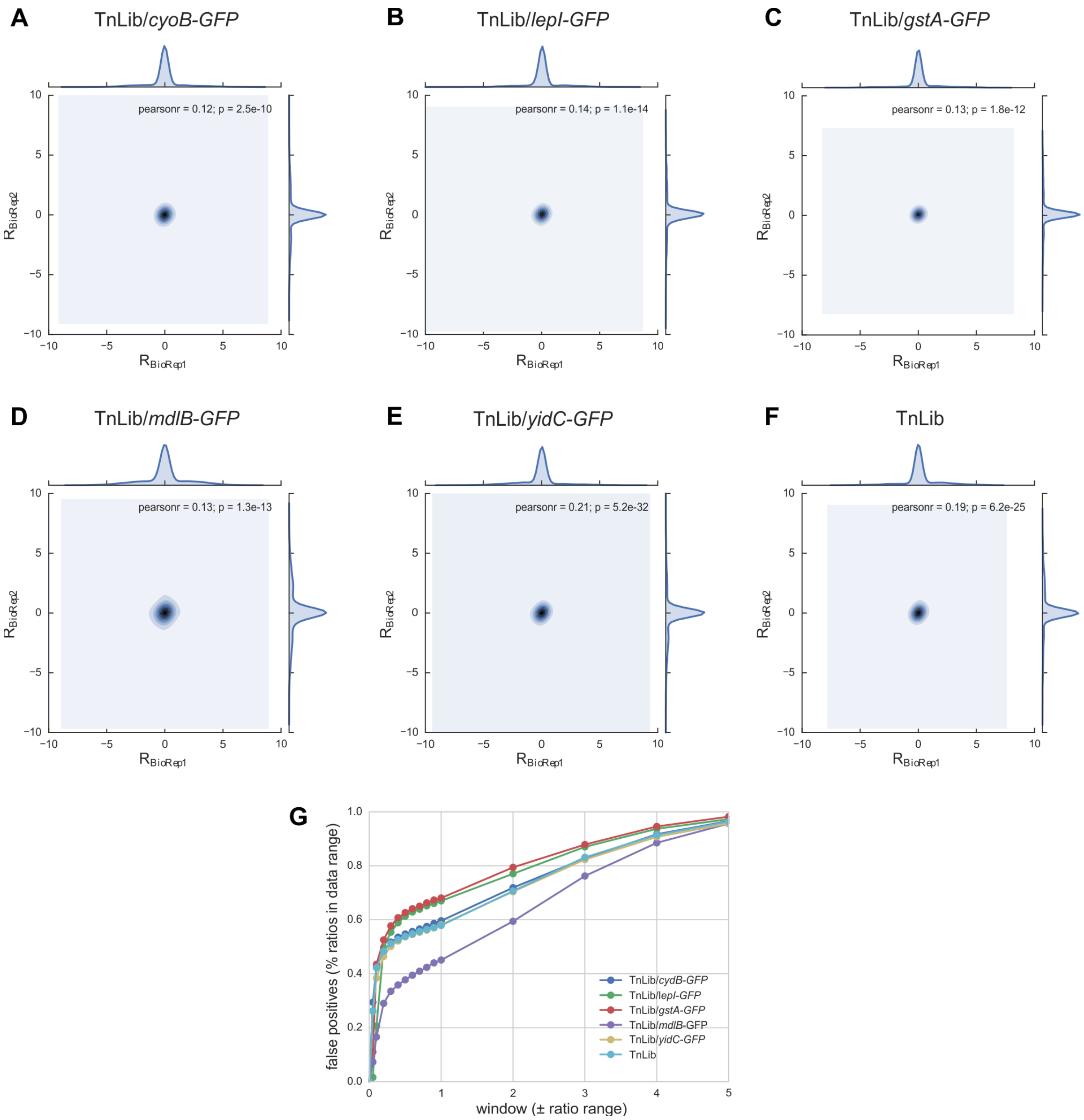

Figure S4. Related to Table S1. Reproducibility of biological replicate enrichment ratios. (A-F) Kernel density estimate analysis was carried out between sorted uninduced biological replicates. We anticipated that there should be no change in enrichment ratio of transposon insertion strains when no expression stress is applied, and thus the majority of ratios should lie at the origin. (G) To approximate a false positive rate of the enrichment ratio, the percent of data outside of the enriched threshold (±2.0) was calculated. We interpret the percent of data outside ±2.0 range as the coefficient of variance for the enrichment ratio and report this percent as the false positive rate.

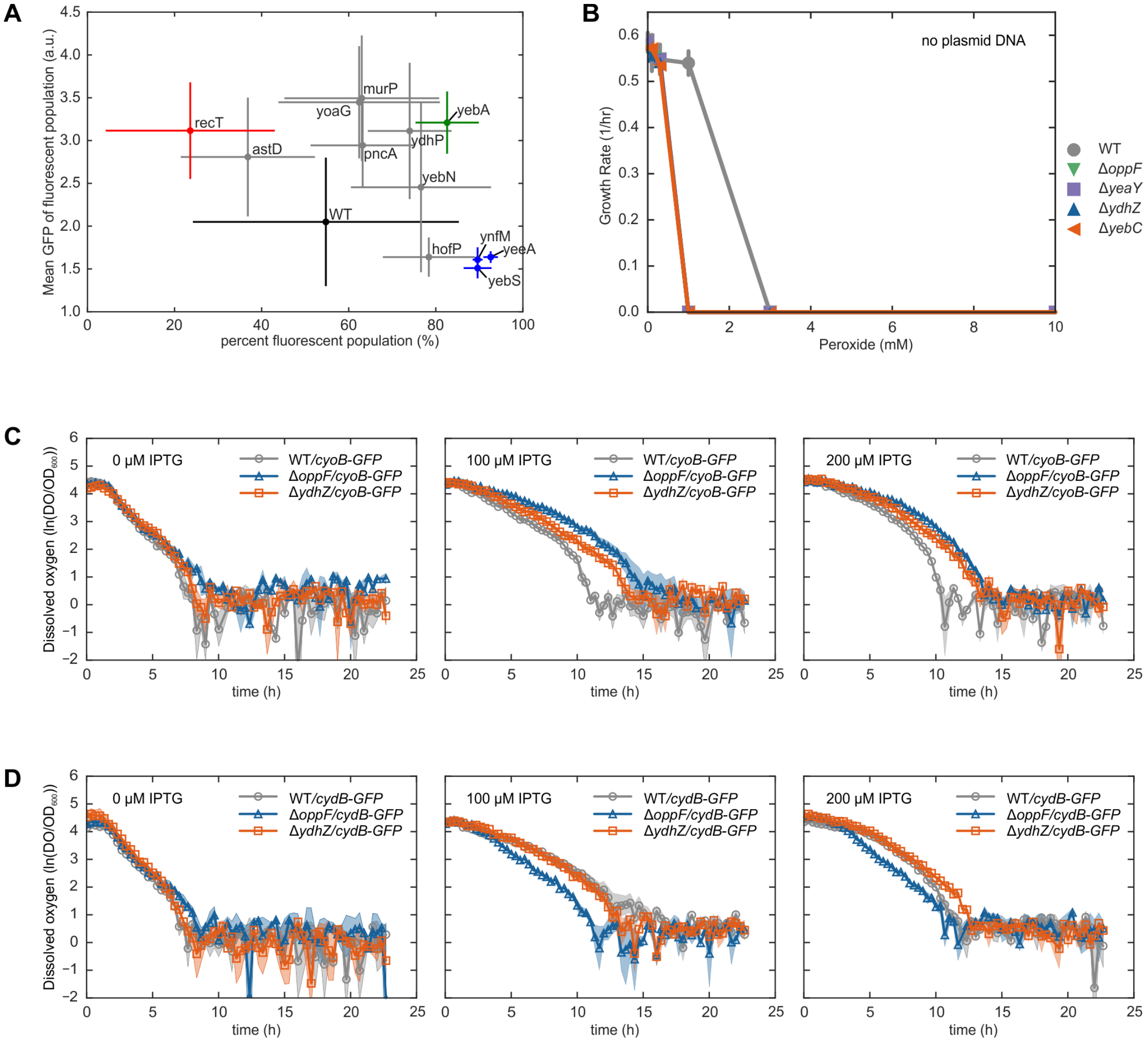

Figure S5. Related to Figure 5 and Table S4. Supplemental data to single gene deletion validation experiments. (A) Strains expressing MdlB-GFP were induced for 4 h and evaluated for IMP-GFP population using flow cytometry (n ≥ 5, error bars represent standard deviation). WT/*mdlB-GFP* (black) showed a large variability in both percent fluorescent population and the mean IMP-GFP signal of that population. Some strains, like Δ*recT/mdlB-GFP* (red), increased the mean IMP-GFP signal but at the cost of lower population of the cells expressing MdlB-GFP. Some strains, like Δ*ynfM*, Δ*yeeA*, and Δ*yebS* (blue), increased the population expressing MdlB-GFP and decreased the variability of how much MdlB-GFP was expressed. Δ*yebA/mdlB-GFP* (green) increased both the percent population expressing MdlB-GFP and the mean MdlB-GFP signal. (B) All non-transformed single gene deletion strains tested for hydrogen peroxide sensitivity are sensitive beyond 1 mM H_2O_2 whereas WT *E. coli* shows sensitivity beyond 3 mM H_2O_2. (C) Dissolved oxygen normalized to cell density for strains expressing the Cyo complex shows that WT uptakes oxygen faster than either Δ*oppF* or Δ*ydhZ*. (D) Dissolved oxygen normalized to cell density for strains expressing the Cyd complex shows that Δ*oppF* uptakes oxygen faster than WT or Δ*ydhZ*.

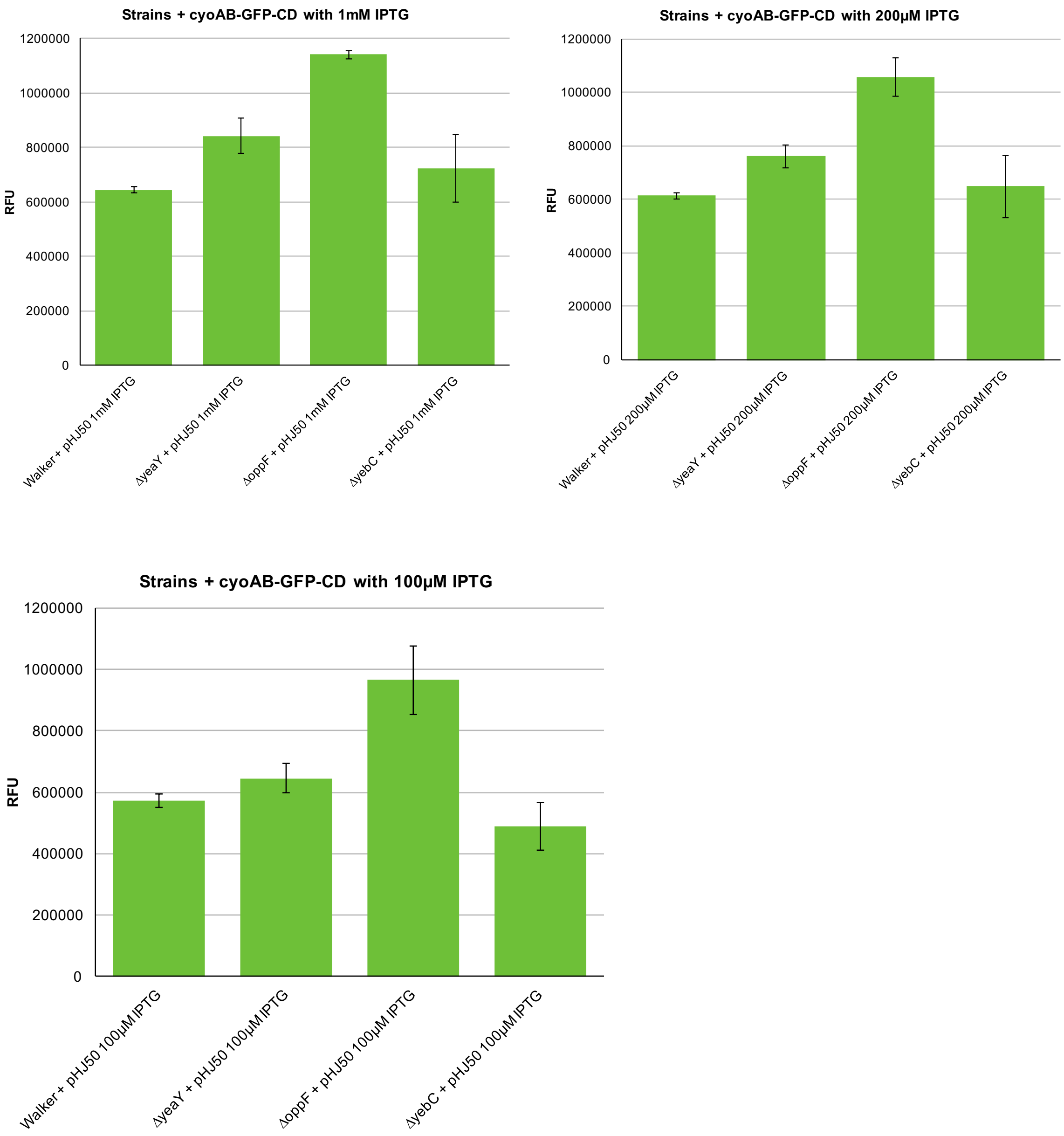

Figure S6. Comparison of the Walker strain with deletion strain discovered in this study. IMP from pHJ50 was used to test the ability for the Δ*oppF*, Δ*yeaY* and Δ*yebC* strain at 100 μM, 200 μM and 1 mM IPTG induction. Relative to the Walker strain C43(DE3) derived from BL21(DE3)[15], a background routinely used for protein expression, the deletion loci tested performed equally well or better.

Supplemental Text.

Calculation of percent membrane protein entries in the Protein Databank (PBD), to-date.
To calculate the percent of PBD entries that are membrane proteins, the total entries for each subcategory below was determined on January 27, 2017 at the following website: http://www.rcsb.org/pdb/results/results.do?qrid=F15AE105&tabtoshow=Current

Of total PBD entries: 3.1%
- Total entries = 126,278
- Membrane protein entries = 2720 α-helical, 864 β-barrel, and 418 monotopic, or 4002 total MPs

Of bacterial PBD entries: 4.4%
- Bacterial entries = 45,569
- Bacterial membrane protein entries = 1474 α-helical, 426 β-barrel, and 111 monotopic, or 2011 total MPs

Of *E. coli* PBD entries: 9.5%
- *E. coli* entries = 8,412
- *E. coli* membrane protein entries = 544 α-helical, 216 β-barrel, and 38 monotopic, or 798 total MPs

Resource List

**Reagent or Resource**	**Source**	**Identifier**
**Chemicals, materials, and recombinant proteins**		
Miller LB	EMD Millipore	1102850500
EZ Rich medium	Teknova	M2105
Kanamycin	Teknova	K2108
Chloramphenicol	Teknova	C0310
IPTG	Sigma-Aldrich	I6758-5G
Q5 DNA polymerase	New England Biolabs	E0555L
Prime Star DNA polymerase	Takara	R050A
Phosphate buffered saline (10x)	Quality Biological	119-069-151
48 well flat bottom plate	BD Falcon	353078
Breathe-easy film	USA Scientific	9123-6100
Biolector FlowerPlate	m2p labs	MTP-48-BOH
Gas impermeable film	Axygen	14-222-873
**Commercial assays**		
Plasmid Miniprep Kit	Qiagen	27106
DNA Clean & Concentrator Kit	Zymo	D4004
Qubit dsDNA HS Assay Kit	ThermoFisher	Q32854
DNEasy Blood and Tissue kit	Qiagen	69506
MiSeq Reagen Kit v2 (50-cycle)	Illumina	MS-102-2001
**Sequence-Based reagents**		
Primers used in this study are listed in Table S5	This study	n/a
**Deposited data**		
Log2 fitness data	This study	GSE95857
**Software and algorithms**		
J5	(45)	j5.jbei.org
FEBA	(27)	bitbucket.org/berkeleylab/feba
Python programming language	n/a	python.org
Circos	(47)	circos.ca
